# Polysomnographic insights into the attention-deficit/hyperactivity disorder and obstructive sleep apnea connection in children

**DOI:** 10.3389/frsle.2024.1451869

**Published:** 2024-10-07

**Authors:** Olga Lacki, James Slaven, Jerry Rushton, Harish Rao, Rohan Thompson, Hasnaa Jalou, Anuja Bandyopadhyay

**Affiliations:** ^1^Department of Pediatrics, Indiana University School of Medicine, Indianapolis, IN, United States; ^2^Department of Biostatistics and Health Data Science, Indiana University School of Medicine, Indianapolis, IN, United States; ^3^Department of Pediatric Pulmonology, Allergy, and Sleep Medicine, Indiana University School of Medicine, Indianapolis, IN, United States

**Keywords:** attention-deficit/hyperactivity disorder, obstructive sleep apnea, sleep disordered breathing, pediatrics, insomnia

## Abstract

**Introduction:**

There is a high prevalence of sleep disturbances and disorders such as obstructive sleep apnea (OSA) in children with attention-deficit hyperactivity disorder (ADHD), though this relationship remains poorly characterized by objective measures. Polysomnographic studies (PSGs) on sleep disruptions in ADHD have yielded inconsistent results. Few have studied polysomnograms in stimulant-medicated vs. non-medicated children with ADHD+/-OSA. This study aimed to elucidate pathognomonic polysomnographic sleep disturbances in children diagnosed with ADHD+/-OSA.

**Methods:**

Medical charts and polysomnograms were retrospectively reviewed for children ages 4-18 who underwent overnight polysomnography at a tertiary care center from 2019 to 2022. ADHD diagnosis was determined by ICD code F90, and OSA was defined by apnea-hypopnea indices (AHI) ≥5 events/hour. Four groups were evaluated: children without OSA or ADHD, children with OSA alone, children with ADHD alone, and children with ADHD+OSA. Statistical analyses identified significant differences among variables of interest.

**Results:**

4,013 children met the study criteria. 2,372 children were without OSA and without ADHD (59.1%), 1,197 with OSA alone (29.8%), 333 with ADHD alone (8.3%), and 111 with ADHD and OSA (2.8%). Insomnia (ICD code G47.00) was prevalent in children with ADHD alone. However, they exhibited significantly better sleep efficiency (SE), than children with OSA alone, and SE did not significantly differ from the other groups. No significant difference in periodic limb movements (PLMs) was found across all groups. The above results held true even after correcting for stimulant prescription.

**Conclusion:**

The increased frequency of clinical insomnia diagnoses in children with ADHD is not associated with any traditional parameters on polysomnogram. Innovative subclinical polysomnographic biomarkers are needed to identify sleep characteristics unique to ADHD. In children with both ADHD and OSA, PSG results do not reveal any distinctive sleep disturbances which cannot be better explained by OSA alone. These findings underscore the importance of screening for OSA in patients with ADHD and clinically assessing for other sleep concerns.

## Introduction

Attention-deficit/hyperactivity disorder (ADHD) affects an estimated 6 million children (9.8% of those aged 3 to 17 in the United States) (Bitsko et al., 2022). Between 25 and 50% of individuals with ADHD report sleep problems (Corkum et al., 1998; Fisher et al., 2014). Some sleep disturbances may be attributed to medications used for treating ADHD, while others are linked to comorbid primary sleep disorders (Gruber, 2009). Primary sleep disorders can lead to sleep restriction, sleep fragmentation, or affect daytime behavior, attention, mood, and physical wellbeing. Many comorbid sleep disorders often go unnoticed and untreated in ADHD populations due to overlap of symptoms (Wajszilber et al., 2018). A recent study revealed that 65% of children with ADHD had a sleep disorder, in contrast to only 17% of controls (Joseph et al., 2022). Despite ADHD's strong association with sleep disorders, particularly obstructive sleep apnea (OSA), the precise links between their sleep disturbances remain unclear.

The incidence of sleep-disordered breathing in ADHD, including OSA, is estimated to range between 25 and 57% among children and adolescents (Sedky et al., 2014). Children with OSA are also disproportionately diagnosed with ADHD, raising the question of whether children with OSA are predisposed to ADHD or if their symptoms result from their sleep-disordered breathing (Chervin et al., 2002). A 2011 literature review indicates that attentional deficits have been reported in up to 95% of patients with OSA. Additionally, six interventional studies have reported improvements in ADHD symptoms following appropriate treatment of OSA (Youssef et al., 2011).

Despite the prevalence of sleep disturbances, objective sleep parameters associated with ADHD remain poorly understood. Two sleep assessments often performed in children are actigraphy studies, featuring a watch-like device measuring movement and light, and polysomnograms (PSGs), which collect additional data on brain wave activity, cardiac activity, eye and limb movement, and respiratory physiology. Sleep efficiency, the ratio between the time a person spends asleep and the total time in bed dedicated to sleep, is one parameter frequently studied in patients with ADHD. Home actigraphy studies suggest increased prevalence of insomnia, characterized by increased sleep latency onset and decreased sleep efficiency in children with ADHD; however, in-lab pediatric sleep studies or PSGs often yield inconsistent results (Liang et al., 2023).

Restless legs syndrome (RLS), diagnosed by clinical criteria, is another sleep disorder which has been linked to ADHD (Migueis et al., 2023) and it shares pathophysiology with periodic limb movement syndrome (PLMS). Research exploring the association between ADHD, RLS, and PLMS has yielded variable results (Fulda and Miano, 2023). Moreover, a recent study revealed that while 81.1% of children with ADHD voiced restless sleep prior to objective testing, only 9.1% of those were diagnosed with restless sleep disorder (RSD) following PSG, with a majority of their restless sleep being actually attributed to other sleep disorders, psychiatric conditions, or effects of medication (Kapoor et al., 2021).

Variances across studies of ADHD and sleep may be in part due to methodological issues, such as small sample sizes and differences in definitions and measurements (Stein et al., 2012). For example, there is significant variability in the AHI cut-off used to characterize OSA in the few studies on ADHD and sleep-disordered breathing, making it difficult to compare their results. Some studies used an AHI cut-off of ≥5, others used ≥1, and some even used ≥2 (Sedky et al., 2014; Bixler et al., 2016).

Some studies suggest that stimulant usage negatively affects sleep (Doghramji et al., 2018), though this has not been studied extensively in children with ADHD. Most studies on PSG parameters do not differentiate between stimulant-medicated and non-medicated patients, and even fewer explore the effects of medication on sleep studies performed in children with both OSA and ADHD. Furthermore, subjects undergoing pharmacologic treatment for ADHD are often excluded from studies (Cortese et al., 2009). Only 3 out of 45 studies in a recent meta-analysis on ADHD and children's sleep examined both medicated and unmedicated ADHD patients and sleep parameters (Liang et al., 2023).

Some studies suggest the existence of distinct phenotypes of sleep disturbances among patients with ADHD who report sleep problems. In 2019, Miano et al. proposed five hypothesized sleep phenotypes in ADHD: narcoleptic-like, delayed sleep onset insomnia, OSA, RLS/PLMS, and sleep EEG epileptiform discharges (Miano et al., 2019). One study examining PSG findings in adolescents with and without ADHD, as well as OSA, observed that patients with ADHD alone exhibited a higher prevalence of PLMs compared to those with both ADHD and OSA. Moreover, individuals with OSA and ADHD experienced an increased frequency of awakenings compared to those with ADHD alone, indicating potential differences in sleep disturbance phenotypes between these groups (Puzino et al., 2022).

Our aim was to identify polysomnogram biomarkers in children with ADHD and OSA. We hypothesized that, when correcting for comorbid OSA in children with ADHD, there would be difference in biomarkers in children with ADHD-alone vs. those with both ADHD and OSA. This would be consistent with the theories of heterogeneous sleep phenotypes in children with ADHD. Specifically, we hypothesized that children with ADHD alone would have higher PLMs, decreased sleep efficiency, and increased sleep fragmentation as measured by arousal indices (mean number of awakenings per hour) compared to those in children with both OSA and ADHD. Appropriate differentiation of ADHD-specific sleep characteristics from those of overlapping disorders such as OSA may aid in early detection and tailored interventions in these vulnerable populations.

## Methods

Medical charts and sleep studies were retrospectively reviewed for 4,013 children ages 4 to 18 who underwent overnight polysomnography following sleep concerns at Riley Hospital for Children from 2019 to 2022. The age cutoff was placed at a minimum of 48 months, as the AAP (American Academy of Pediatrics) guidelines for ADHD diagnosis are described in children 4 years and older (Subcommittee on Attention-Deficit/Hyperactivity Disorder et al., 2011). For patients who had multiple sleep studies obtained during this time, only the first sleep study was taken into consideration. Sleep studies of all patients were included in this research, with the exclusion criteria of an age < 48 months. The study was approved by the Institutional Review Board approval to maintain a research database on all patients undergoing polysomnographic sleep studies.

### Variables

Demographic data was collected and included: date of birth, age at time of PSG, gender, and BMI. Data on medical comorbidities was collected from diagnostic billing codes placed by clinicians (ICD-10). Comorbidities studied were obesity, ADHD, and insomnia. Information about usage of medications known to influence sleep at the time of study was collected from the charts of ADHD+ patients by same-day questionnaire, or if not available, by documentation of medication use in chart within 2 months from date of sleep study as well as prescription fill date. These included stimulant medications, serotonin-norepinephrine reuptake inhibitors (SNRIs), selective-serotonin reuptake inhibitors (SSRIs), benzodiazepines, antiepileptics, melatonin, clonidine, and guanfacine. Patients with adenotonsillar hypertrophy, neuromuscular disorders, history of prematurity, or craniofacial abnormalities were not excluded to preserve representation of the heterogeneous population observed in clinical practice.

### Polysomnography

Children underwent PSG at an accredited sleep laboratory as part of clinically indicated care. PSG tests were performed in technical accordance with standards proposed by the American Academy of Sleep Medicine (AASM), by registered polysomnography technicians. Electroencephalogram (placement of frontal [F3,F4], central [C3,C4], occipital [O1,O2] electrodes referenced to the opposite mastoid electrodes [M1,M2]), electro-oculogram, electromyogram (chin and both legs), electrocardiogram, pressure transducer and thermistor airflow, uncalibrated respiratory inductance plethysmography, oximetry, and end-tidal CO2 (ETCO2) data with video monitoring of the study for scoring support was collected. All studies were scored by a pediatrician board certified in sleep medicine, in accordance with the pediatric scoring rules proposed by the AASM (Berry et al., 2012).

The standard PSG collects a number of variables as a multi-parametric test to evaluate and diagnose sleep disorders. PSG data collected and analyzed included: date of study, age at time of study, percentage of sleep spent in the rapid-eye movement stage of sleep (REM), percentage of sleep spent in stages N1 and N2, percentage of sleep spent in slow wave sleep (N3), sleep efficiency, arousal index, apnea hypopnea index (AHI), total periodic limb movement index (PLM-I), average oxygen saturations in sleep, and average ETCO2 during sleep.

### Definitions

Pediatric OSA is defined as the presence of snoring, labored breathing, obstructive events, or daytime consequences (sleepiness, hyperactivity), along with 1 or more polysomnography (PSG) findings. These PSG findings include (1) ≥1 significant obstructive event (obstructive or mixed apnea, or obstructive hypopnea) affecting at least 2 consecutive breaths per hour of sleep, or (2) obstructive hypoventilation manifested by peripheral arterial carbon dioxide (PaCO2) >50 mm Hg for >25% of sleep time, coupled with snoring, paradoxical thoracoabdominal movement, or flattening of the nasal airway pressure waveform (Ehsan and Ishman, 2016).

There is considerable variation in the AHI cut off utilized for diagnosing children with OSA. The AASM Manual for the Scoring of Sleep and Associated Events classifies an AHI ≥1 and < 5 as mild OSA, AHI ≥ 5 and < 10 as moderate OSA, and AHI ≥ 10 classifies severe OSA (Berry et al., 2017). In our study, clinically significant OSA was defined by an apnea hypopnea index (AHI) of ≥5 events/hour (Ishman et al., 2023). The same analyses were conducted with an AHI threshold lowered to ≥1 event/h to identify any differences based on different thresholds. An elevated PLM index was defined as a PLM index >5/h.

### Statistics

The PSG parameters and demographic variables of interest in children with and without ADHD, as outlined above, were compared. Utilizing the AHI results from PSGs, these groups were further stratified to evaluate the following cohorts of interest: Children with ADHD+/OSA-, ADHD+/OSA+, ADHD-/OSA+ and ADHD-/OSA-. Children who underwent PSG but did not have ADHD and/or OSA (ADHD-/OSA-) acted as a control group in this study. Although these patients did report sleep concerns leading to a sleep study, in the absence of normative reference data in our particular population, this group acted as a proxy for comparison.

Relevant analyses were performed to determine if there were significant differences amongst the variables of interest between the various groups. Continuous variables were analyzed using Student's *t*-tests for two group comparisons and Analysis of Variance (ANOVA) models for >2 groups. Categorical variables were analyzed using Chi-Square tests, and Fisher's Exact was used when 25% or more of the cells had expected cell counts < 5. All analytic assumptions were verified, with transformations being used where possible and the Wilcoxon-Kruskal-Wallis non-parametric test being used where necessary. Although several sub-analyses were performed, no multiple comparison tests were performed, as the clinicians decided to look at overall outcomes and results of the analyses. Analyses were performed using SAS v9.4 (SAS Institute, Cary, NC). Values were reported as means (standard deviations) for continuous variables and frequencies (percentages) for categorical variables, with *p*-values from ANOVA and Chi-Square tests, respectively. *P*-value superscripts were labeled to represent the following: a = Both groups with ADHD significantly differed, b = ADHD+/OSA- vs. ADHD-/OSA+ significantly differed, c = ADHD+/OSA- vs. controls significantly differed, d = both groups with OSA differed, e = ADHD+/OSA+ vs. controls significantly differed, and f = both groups without ADHD significantly differed. Missing superscripts indicated that no pairwise comparisons were significantly different with a Bonferroni adjustment.

## Results

### Demographics and diagnoses

#### ADHD+ vs. ADHD-

Of the 4,013 children reviewed, 444 children had a diagnosis of ADHD (11%) (Table 1) made by their primary care provider or a subspecialist. 1,308 children, both with and without ADHD, were diagnosed with clinically significant OSA (AHI ≥5) based on their PSG (32.6%). The mean age of children with ADHD was 14.7 months older than those without ADHD. Both groups presented with more males than females, with a higher gender disparity seen in children with ADHD than those without ADHD. 25% of the children with ADHD met criteria for clinically significant OSA (AHI ≥5). 70% of the children with ADHD had an AHI ≥1. Children with ADHD were more frequently diagnosed with insomnia than those without ADHD.

**Table 1:** Demographics and diagnoses.

	**ADHD**		**No ADHD**		***p*-value**
*N=*	444		3,569		
Mean age	10.4 yrs		9.1 yrs		< 0.0001
Male	149 (33.6%)		1,623 (45.5%)		< 0.0001
Female	295 (66.4%)		1,946 (54.5%)		
BMI	23.5 (9.1)		24.8 (25.2)		0.2867
Insomnia dx	116 (26.1%)		253 (7.1%)		< 0.0001
Prematurity dx	21 (4.7%)		107 (3.0%)		0.0472
	**ADHD**+**/OSA-**	**ADHD**+**/OSA**+	**ADHD-/OSA**+	**ADHD-/OSA-**	
*N=*	333	111	1,197	2,372	
Mean age	10.0 yrs	12.0 yrs	9.5 yrs	8.9 yrs	< 0.0001^acdef^
Male	111 (33.3%)	38 (34.2%)	513 (42.9%)	1,110 (46.8%)	< 0.0001
Female	222 (66.7%)	73 (65.8%)	684 (57.1%)	1,262 (53.2%)	
BMI	21.6 (7.5)	29.3 (10.8)	27.1 (22.2)	23.5 (26.7)	0.0002^abf^
Insomnia dx	96 (28.8)	20 (18.0)	57 (4.8)	196 (8.3)	< 0.0001

Superscript “a” refers to ADHD+/OSA- vs. ADHD+/OSA+; “b” to ADHD+/OSA- vs. ADHD-/OSA+; “c” to ADHD+/OSA- vs. ADHD-/OSA-; “d” to ADHD+/OSA+ vs. ADHD-/OSA+; “e” to ADHD+/OSA+ vs. ADHD-/OSA-; and “f” to ADHD-/OSA+ vs. ADHD-/OSA-.

#### ADHD+/OSA-, ADHD+/OSA+, ADHD-/OSA+, and ADHD-/OSA-

**Figure 1:**
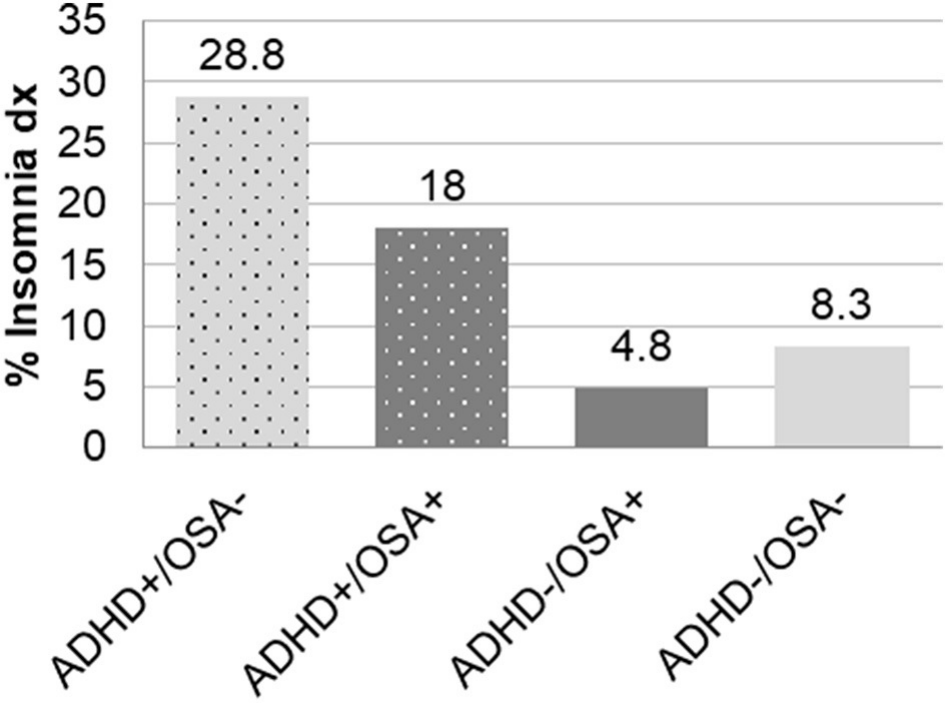
Frequency of insomnia diagnoses in children with and without ADHD or OSA demonstrating significant differences between all six pairwise comparisons: ADHD+/OSA- vs. ADHD+/OSA+, ADHD+/OSA- vs. ADHD-/OSA+, ADHD+/OSA- vs. ADHD-/OSA-, ADHD+/OSA+ vs. ADHD-/OSA+, and ADHD+/OSA+ vs. ADHD-/OSA-.

As seen in Figure 1, the children were divided into the four groups of interest in regards to ADHD and OSA: 333 children (8.3%) had ADHD only, 111 children (2.8%) had both ADHD and OSA, 1,197 children had OSA only (29.8%), and 2,372 children (59.1%) did not have OSA nor ADHD. The ADHD-/OSA- group acted as a control for comparisons. Children in the ADHD+/OSA- group were younger than those in the ADHD+/OSA+ group by a mean of 24.6 months. The ADHD+/OSA- group had a lower mean BMI as compared to the ADHD+/OSA+ and ADHD-/OSA+ groups.

The ADHD+/OSA- group had an increased prevalence of insomnia diagnoses compared to those in the ADHD+/OSA+ group, though the ADHD+/OSA+ group demonstrated an increased prevalence of insomnia diagnoses compared to both groups without ADHD (Table 1, Figure 1). There was no significant difference in the number of children prescribed stimulant and psychoactive medications at the time of sleep study in the ADHD+/OSA- and ADHD+/OSA+ groups (Supplementary Table 2)

### Polysomnographic parameters

#### ADHD+ vs. ADHD-

There were no significant differences in the mean arousal index, sleep efficiency, mean PLM index, or number of elevated PLM indices between children with and without ADHD (Table 2). The mean AHI of both groups of children was above 5, meeting criteria for OSA, and the AHI of children with ADHD was lower than that of children without ADHD. The percentage of REM sleep was higher in children with ADHD than those without ADHD (18.2% vs. 16.9%, *p* = 0.0004). However, although these differences in the percentage of REM sleep were statistically significant, they were not clinically significant.

**Table 2:** PSG parameters.

	**ADHD**		**No ADHD**		***p*-value**
Arousal index	15.1 (10.1)		15.5 (11.4)		0.4070
Sleep efficiency	80.1% (12.9)		79.7 (13.0)		0.5541
% REM sleep	18.2 (13.1)		16.9 (6.5)		0.0004
AHI	5.3 (10.4)		7.4 (13.8)		0.0015
Saturation	96.7 (1.4)		96.7 (1.4)		0.9691
CO_2_	43.2 (3.5)		43.7 (3.6)		0.0388
PLM index	2.0 (5.5)		1.7 (5.2)		0.3760
Elevated PLMs	45 (10.1%)		325 (9.1%)		0.4797
Stage 1 (%)	18.2 (16.9)		19.9 (18.0)		0.0557
Stage 2 (%)	47.9 (10.3)		45.3 (9.9)		< 0.0001
Stage 3 (%)	28.9 (9.8)		30.7 (10.0)		0.0003
	**ADHD**+**/OSA-**	**ADHD**+**/OSA**+	**ADHD-/OSA**+	**ADHD-/OSA-**	
Arousal index	12.2 (5.9)	23.7 (14.3)	22.8 (15.6)	11.8 (5.7)	< 0.0001^abef^
Sleep efficiency	80.6 (12.8)	78.7 (13.2)	77.4 (14.5)	80.9 (11.9)	< 0.0001^bf^
Stage 1 (%)	14.8 (3.9)	28.1 (20.7)	26.9 (21.6)	15.5 (13.7)	< 0.0001^abef^
Stage 2 (%)	47.8 (10.5)	48.0 (10.0)	45.0 (10.3)	45.5 (9.6)	< 0.0001^bcd^
Stage 3 (%)	29.6 (9.4)	26.5 (10.8)	29.6 (10.2)	31.3 (9.8)	< 0.0001^acdef^
% REM sleep	17.8 (12.1)	19.4 (15.8)	16.2 (6.6)	17.3 (6.4)	< 0.0001^bdef^
AHI	1.5 (1.2)	16.5 (16.1)	18.7 (19.4)	1.7 (1.3)	< 0.0001^abef^
Saturation	96.9 (1.3)	96.0 (1.3)	96.1 (1.5)	96.9 (1.2)	< 0.0001^abef^
CO_2_	43.2 (3.6)	43.1 (3.4)	43.8 (3.7)	43.6 (3.5)	0.0465
PLM index	1.8 (5.4)	2.4 (6.0)	1.6 (5.1)	1.8 (5.2)	0.3845
Elevated PLMs	30 (9.0)	15 (13.5)	105 (8.8)	220 (9.3)	0.4304

Superscript “a” refers to ADHD+/OSA- vs. ADHD+/OSA+; “b” to ADHD+/OSA- vs. ADHD-/OSA+; “c” to ADHD+/OSA- vs. ADHD-/OSA-; “d” to ADHD+/OSA+ vs. ADHD-/OSA+; “e” to ADHD+/OSA+ vs. ADHD-/OSA-; and “f” to ADHD-/OSA+ vs. ADHD-/OSA-.

#### ADHD+/OSA-, ADHD+/OSA+, ADHD-/OSA+, and ADHD-/OSA-

**Figure 2:**
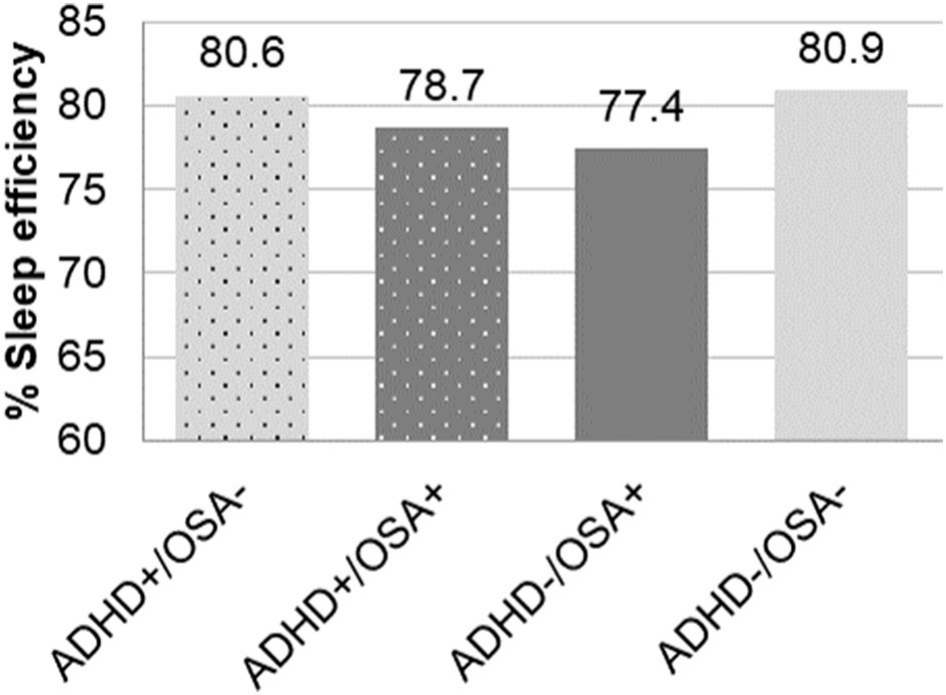
Percent of sleep efficiency in children with and without ADHD or OSA demonstrating significant differences between ADHD+/OSA- vs. ADHD-/OSA+ and ADHD/OSA+ vs. ADHD-/OSA-.

When accounting for both ADHD and OSA, both groups with OSA exhibited greater arousal indices than the groups without OSA, regardless of comorbid ADHD (Table 2). The arousal index of the ADHD+/OSA- group was not significantly higher than the control group. Children of the OSA+ groups, both with and without ADHD, had a significantly decreased sleep efficiency compared to patients without OSA (77.3% vs. 80.7%, *p* < 0.0001). The ADHD+/OSA+ group's sleep efficiency was decreased, though not significantly, from the control group. The ADHD-/OSA+ group did demonstrate a sleep efficiency which was significantly decreased from the control group. However, children of the ADHD+/OSA- group did not demonstrate significantly decreased sleep efficiency compared to any of the other groups; in fact, those with ADHD alone had significantly increased sleep efficiency as compared to children with OSA alone (Table 2, Figure 2).

The ADHD-/OSA- group had the highest amount of slow wave sleep (31.3%), the group with ADHD alone had the same amount of slow wave sleep as the group with OSA alone (29.6%), and the ADHD+/OSA+ group had the least amount of slow wave sleep (26.5%, *p* < 0.0001).The ADHD+/OSA+ group exhibited the highest percentage of REM sleep, while the ADHD+/OSA- group did not significantly differ in percentage of REM from the ADHD-/OSA- group (Table 2). Once again, these differences in percentage of REM sleep were significant statistically, though not clinically.

In comparison to the children in ADHD+/OSA- group with a stimulant prescription, there was an insignificant trend toward improved sleep efficiency in ADHD+/OSA- children without stimulant prescriptions (Tables 3A, B). Children of the ADHD+/OSA- group without stimulant prescriptionsalso had an increased percentage REM sleep compared to those with stimulants, though this also was not a significant result. Among the children with ADHD, both with and without comorbid OSA, there was no significant difference in any sleep stage, including slow wave sleep or REM sleep, between those who were not prescribed stimulants and those who were. There was no significant difference or remarkable trends among the polysomnographic parameters between children of the ADHD+/OSA+ group with and without stimulant prescription.

**Table 3A:** PSG sleep parameters in patients with ADHD without OSA, with and without stimulant prescription.

	**ADHD+/OSA- stimulant +**	**ADHD+/OSA- stimulant -**	***p*-values**
*N*=	140	193	
Arousal index	12.6 (5.8)	11.8 (6.0)	0.2316
Sleep efficiency	79.0 (13.0)	81.7 (12.5)	0.0601
Stage 1 (%)	14.9 (14.0)	14.8 (13.9)	0.9363
Stage 2 (%)	48.5 (10.6)	47.3 (10.4)	0.3047
Stage 3 (%)	29.9 (9.6)	29.5 (9.2)	0.6680
% REM sleep	16.4 (9.8)	18.8 (13.5)	0.0755
AHI	1.5 (1.1)	1.5 (1.2)	0.5255
Average saturations	96.9 (1.2)	97.0 (1.3)	0.4678
Average CO_2_	43.4 (3.8)	43.1 (3.4)	0.5165
PLM index	1.6 (4.5)	2.0 (5.9)	0.4613
Elevated PLM index	14 (10.0)	16 (8.3)	0.5906

**Table 3B:** Sleep parameters in patients with ADHD and OSA, with and without stimulant prescription.

	**ADHD+/OSA+ stimulant +**	**ADHD+/OSA+ stimulant -**	***p*-values**
*N*=	52	59	
Arousal index	24.5 (15.3)	23.0 (13.5)	0.6091
Sleep efficiency	79.1 (14.5)	78.3 (12.0)	0.7230
Stage 1 (%)	30.4 (20.1)	26.1 (21.2)	0.2701
Stage 2 (%)	48.5 (8.0)	47.6 (11.6)	0.6078
Stage 3 (%)	25.2 (7.9)	27.7 (12.8)	0.1976
% REM sleep	19.7 (15.5)	19.1 (16.1)	0.8541
AHI	17.0 (19.8)	16.0 (12.0)	0.8020
Average saturations	96.1 (1.3)	95.9 (1.4)	0.3444
Average CO_2_	43.6 (3.9)	42.7 (2.9)	0.2796
PLM index	2.4 (6.4)	2.5 (5.7)	0.8889
Elevated PLM index	6 (11.5)	9 (15.3)	0.5417

Children in ADHD+/OSA- group prescribed SSRI/SNRI medications showed a non-significant decrease in REM percentage, compared to those without the medications (Supplementary Table 3A). The arousal index was higher in the ADHD+/OSA- group prescribed SSRI/SNRIs than those not prescribed SSRI/SNRIs. There was no significant difference in the arousal index or percent of REM sleep in children in the ADHD+/OSA+ group, regardless of SSRI/SNRI prescription (Supplementary Table 3B).

## Discussion

In this large, retrospective cross sectional study of over 4,000 children who reported sleep concerns, we did not find any significant decrease in sleep efficiency or increase in sleep fragmentation and limb movements in children with ADHD alone, after correcting for comorbid OSA. Interestingly, our results demonstrated that children with ADHD had a high prevalence of insomnia diagnoses by ICD code. Although stimulant prescription was not associated with increased sleep fragmentation in children with ADHD, SSRI/SNRI prescription was associated with increased arousal indices. This study highlights the importance of clinical assessment for comorbid sleep disorders and judicious use of medications which can alter sleep architecture in children with ADHD.

### Sleep efficiency and insomnia

In our study, we found an increased prevalence of insomnia diagnoses by ICD code prior to PSG in children with ADHD compared to children without ADHD. Despite this, children with ADHD did not show any objective decrease in sleep efficiency or increase in sleep fragmentation (as noted by arousal index) on PSG.

Children with ADHD without comorbid OSA had the highest rate of insomnia diagnoses prior to PSG at 28%. However, their sleep efficiency, our proxy for insomnia, during in-lab PSGs was similar to children without ADHD. Children with both ADHD and OSA also had an increased rate of insomnia diagnoses as compared to both groups with OSA, though lower than those with ADHD alone. This finding could be partially explained by physician bias, when insomnia may not be diagnosed as a comorbidity if clinical symptoms of OSA are considered a more significant factor contributing to sleep disturbance. Only the groups with OSA, irrespective of comorbid ADHD, showed decreased sleep efficiency and an increased mean sleep fragmentation compared to the control group. The inconsistency in reported differences in sleep efficiency among patients with ADHD in previous studies may partially stem from the lack of control for potential comorbid OSA. To elaborate, one 2009 meta-analysis, which concluded that children with ADHD had decreased sleep efficiency, also found a significant positive association between ADHD and higher AHI (Cortese et al., 2009).

There are significant challenges of using sleep efficiency as a proxy for insomnia. In sleep studies which are measured over several days, there is a well-known phenomenon known as the “first night effect” wherein the first night of PSG recording is associated with decreased total sleep time, decreased sleep efficiency, and decreased REM sleep (Agnew et al., 1966). Thus, a PSG which only takes place over one night may demonstrate sleep quality worse than the patients' baseline. One also cannot account for night-to-night variability of patient's sleep with only a single night of data. While some studies have suggested that children with ADHD have higher night-to-night variability in their sleep quality than their typically developing peers, this finding has been controversial in the literature (Poirier and Corkum, 2018).

Our PSGs, and the majority of PSGs in children and adolescents in the relevant literature, took place in a sleep laboratory (Liang et al., 2023). It may be possible that several nights of home actigraphy, an instrument measuring light and movement, may be a better representation of patients' environment at home and hence be an accurate marker of patients' night-to-night sleep quality. It may also be possible that several nights of home actigraphy, an instrument measuring light and movement, may be a better representation of patients' baseline sleep duration at home than a single night of an in-laboratory PSG study. Some research suggests that an aggregation of five or more nights of measurement via actigraphy is needed to obtain a reliable measure [of baseline sleep duration] (Acebo et al., 1999). However, there are variables present at home which are not as influential in a standardized sleep laboratory such as inadequate sleep hygiene, environmental and familial stressors, and easier access to distractors such as technology. Therefore, while home actigraphy studies may provide valuable data regarding the patients' sleep patterns, they do not provide as much insight into whether the sleep disturbances are mainly due to external environmental factors or whether there is an innate physiologic basis for them. Additionally, standard actigraphy studies do not provide data on AHI, thus, comorbid OSA may be a major factor in reported sleep disturbances. This is particularly important as sleep efficiency is more likely to be less in ADHD populations on actigraphy studies compared to outcomes of PSG studies (Liang et al., 2023).

Another reason why children with ADHD may demonstrate clinical symptoms of insomnia outside of the sleep laboratory in more familiar surroundings, such as at home, may have to do with their internally driven sleep-wake cycles. Patients with ADHD are thought to be predisposed to circadian rhythm disorders such as delayed sleep phase (Snitselaar et al., 2017). In the context of bedtimes and school hours, these children may exhibit sleep deprivation. However, when evaluated on a polysomnogram with a standardized start time, they may be able to demonstrate adequate sleep efficiency.

It is also possible that the normal sleep efficiency demonstrated in our PSG study among a population of children with ADHD frequently diagnosed with insomnia could be explained by paradoxical insomnia. Paradoxical/subjective insomnia, previously known as sleep state misperception, is a condition wherein individuals perceive themselves to have insomnia despite experiencing a normal sleep pattern on objective measures of sleep (Edinger and Krystal, 2003). The diagnosis of insomnia by primary care physicians often relies on subjective reports of sleep disturbances from parents and children, potentially diverging from objective findings (Rezaie et al., 2018). Notably, sleep efficiency does not differentiate between delayed onset insomnia and sleep maintenance insomnia, as both could lead to decreased sleep efficiency.

Finally, in our study, it is crucial to acknowledge that children with OSA, both with and without ADHD, did manifest decreased sleep efficiency compared to those with ADHD alone and those without either condition. This highlights that traditional PSG variable of sleep efficiency is strongly associated with OSA and may not be a useful predictor of insomnia in children with ADHD. Perhaps there are unique sleep disturbances present in children with ADHD which cannot be consistently perceived by the standard polysomnogram.

### Periodic limb movements and restless legs syndrome

Periodic limb movements (PLMs) are thought to be strongly correlated with the clinical diagnosis of restless legs syndrome (Stefani et al., 2017). Some studies report increased rates of restless legs syndrome and PLMs in children with ADHD (Snitselaar et al., 2017). Our study did not observe elevated PLMs in children with ADHD–there was no significant difference or trend in the mean PLM indices or percent of elevated PLMs across all study groups. In contrast, a 2022 research study found that adolescents (mean age 16.4 years) with ADHD alone showed higher PLM indices (6.0 +/-0.8) than patients with ADHD and OSA (4.0 +/−0.9), or those without either condition (3.3 +/−0.4) (Puzino et al., 2022). In comparison, our study had a younger population (9.9 years) and overall had a much lower incidence of PLMs across all groups. There may be inter-center variability in the data collection and scoring of limb movement. Alternatively, these discrepancies may be suggestive of a different phenotype of ADHD-PLMD which can be seen in older children.

An earlier study researching night-to-night variability in PLMs concluded that a single-night study is sufficient to confirm PLMs (Sforza and Haba-Rubio, 2005). It has been frequently reported that patients with ADHD have increased variability in their sleep from night to night, but those findings were inconsistent, and a more recent actigraphy study did not suggest that children with ADHD had greater variability than their peers (Poirier and Corkum, 2018). However, other studies continue to demonstrate high night-to-night variability in PLM indices, with some suggesting that the more stable periodicity index should be used in place of PLM indices (Skeba et al., 2016). In fact, it has been proposed that PLMs in children with ADHD are often not identified on traditional PSGs because of their low periodicity (Ferri et al., 2013).

### Medication use and sleep study parameters

Children with ADHD prescribed stimulant medications did not demonstrate worse sleep efficiency or elevated arousal indices than those not prescribed stimulants. Stimulant use has been linked to worsening insomnia and sleep quality (Doghramji et al., 2018). Although evidence suggests that stimulants may disrupt sleep in individuals with ADHD (Ironside et al., 2010; Spruyt and Gozal, 2011), clinical observations also indicate paradoxical effects of stimulants (Hvolby, 2015). These effects can lead to symptom alleviation, calming patients, and facilitating sleep (Jerome, 2001; Kratochvil et al., 2005). Moreover, to mitigate potential symptom rebound as drug concentrations decrease, administering an additional dose of a short-acting stimulant or opting for a formulation with an extended duration of action may prevent sleep disturbances linked to worsening hyperactivity or behavioral challenges at bedtime (Carlson and Kelly, 2003; Cortese et al., 2013).

Our study demonstrated increased arousal index (13.6/h) and thereby suggested worsened sleep fragmentation in children with ADHD (without comorbid OSA) on SSRI/SNRI. This is consistent with reports from adult literature (Zhou et al., 2023). This highlights the importance of assessing subjective perception of sleep quality in children with ADHD on these medications.

Due to the overlap in daytime symptomatology between children with ADHD and children with OSA, and the unfortunate underdiagnosing of OSA in children (Erichsen et al., 2012), it is important to note the effects of stimulant medication on OSA sleep parameters. Our results indicate that children with both ADHD and OSA who are prescribed stimulant medications do not demonstrate decreased AHI or improved oxygen saturation compared to their untreated peers. This population is potentially vulnerable to the long-term consequences of their apneas if their daytime symptoms are masked by stimulants and their OSA goes undiagnosed and untreated (Gozal et al., 2008; Chan and Artin Li, 2024).

### Gender differences

In this study, males were overrepresented compared to females across all four groups. The group with the largest difference between males and females was the group with an ADHD diagnosis. This is consistent with the current literature on gender differences in ADHD, showing that male children with ADHD are more likely to be diagnosed than females with ADHD (Willcutt, 2012). Literature investigating the incidence of ADHD in clinical populations vs. the general population demonstrated that this effect is due to referral bias, and not objective measures, in part due to the higher incidence of inattentive symptoms in females (Rucklidge, 2010). Unfortunately, this likely means that many female children may be undiagnosed with ADHD or not diagnosed until later in life (Fraticelli et al., 2022).

There are also significant gender differences between males and females in screening, diagnosis, and treatment of sleep-disordered breathing. Among adults, for example, women are less likely to be screened for OSA, referred for a sleep study, and receive treatment, despite similar symptoms to their counterparts (Lindberg et al., 2017). While some studies suggest increased prevalence in OSA in post-pubertal males, overall evidence for a difference in OSA prevalence based on gender in children and adolescents is not conclusive (Brockmann et al., 2017). While some research suggests that females in adolescence and pre-adolescence have more impaired sleep than their counterparts, more studies are needed in the younger population to elucidate whether there is a difference in quality of sleep between different genders in childhood (Smidt et al., 2022).

### Strengths and limitations

Our study is the largest single-center report on sleep study parameters and ADHD in children, thereby increasing its overall strength. This larger sample size also allowed for comparison between children with and without stimulant prescription divided into subgroups. All sleep studies were performed in a single center and scored by sleep physicians with high interscorer reliability. Some of the inconsistencies across studies on OSA and ADHD are thought to be due to nonstandard sleep measures across studies and variability in AHI cutoffs used for the children with OSA (Bixler et al., 2016; Lunsford-Avery et al., 2016). In this present study, we demonstrated the same findings regarding sleep efficiency and PLMs when OSA was defined as an AHI ≥5 events/h (moderate to severe OSA) as when the inclusion criteria was broadened to include those with an ] AHI ≥ 1.

Our study had several limitations due to its retrospective nature. It was not possible to assess the severity of ADHD at the time of sleep study or perform any psychometric or neurobehavioral assessment. Being limited to that which was uploaded to the EMR meant not having data on the severity of the child's ADHD or the sub-type (inattentive, hyperactive-impulsive, or both). Some of these patients were referred from outside primary care physician, thereby retrieving such data was outside the scope of our study. Due to these limitations, we also could not reliably determine subjects' upper airway anatomy at the time of the sleep study, and could not deduce whether their obstructive sleep apnea was due to anatomical obstruction or a low arousal threshold.

Additionally, retrospective EMR data for supplements such as iron and ferrous sulfate was scarce. Iron supplementation has been shown to affect sleep parameters such as periodic limb movements (Dye et al., 2017). Though physicians do sometimes prescribe these supplements, families often buy them over the counter without a prescription. This makes it difficult to deduce whether a child is receiving supplements from the EMR alone, unless a provider explicitly asked and documented it. A prospective study inquiring regarding iron supplementation and checking serum ferritin levels would be better suited to study PLMs in these populations.

Ideally, our study could also include healthy children and children with ADHD from the community without sleep concerns warranting a clinical PSG. Due to the retrospective nature of this study, this was not possible.

### Future directions

Our data suggests that the polysomnographic sleep disturbances such as decreased sleep efficiency often attributed to ADHD may be better explained by comorbid OSA. However, the high incidence of insomnia diagnoses in ADHD patients implies that there may be sleep disturbances linked to ADHD that cannot be adequately evaluated by traditional polysomnography. It is interesting that our children with ADHD alone and those with OSA alone have decreased deep sleep, or slow wave sleep, compared to those without either comorbidity, and those with both ADHD and OSA have decreased deep sleep compared to either factor alone. A recent study of sleep in adolescents with ADHD looked toward a novel parameter collected from EEG data, the odds-ratio-product (ORP), to investigate differences in sleep depth and intensity. The authors report that only stimulant-free adolescents with ADHD had decreased sleep depth in non-REM sleep compared to children taking stimulants and those without ADHD (Ricci et al., 2022). However, their population of stimulant-free adolescents with ADHD also demonstrated significantly increased AHI in comparison to all other groups. Further research into novel parameters investigating sleep depth such as ORP and their utility in children and adolescents with ADHD and without comorbid OSA is warranted.

The International Classification of Sleep Disorders states that physiological abnormalities may exist in the sleep tracing which are too subtle to be detected by current recording methods such as PSG (Sateia, 2014). One unique study utilized a different method—EEG power spectral analysis—to study sleep in ADHD. They found significant differences in the brain wave activity following spindle epochs in the left hemisphere of children with ADHD compared to children without ADHD (De Dea et al., 2018). The field of sleep medicine may look toward utilizing novel methods such as machine learning to analyze the microarchitecture of polysomnographic studies and correlate it to ADHD/comorbid sleep disorder biomarkers.

## Conclusion

There is a high prevalence of comorbid sleep disorders in children with ADHD. The heterogeneity of ADHD sleep-related concerns makes it difficult to delineate what is primary ADHD vs. ADHD symptomatology secondary to an undiagnosed primary sleep disorder. Children with ADHD, even those without comorbid OSA, have a higher frequency of insomnia diagnoses by subjective measures. However, they do not have worse sleep efficiency or increased sleep fragmentation on polysomnogram unless they have comorbid OSA. In fact, in children with ADHD and OSA, traditional polysomnogram parameters do not reveal any unique sleep disturbances which cannot be better explained by OSA alone. This underscores the importance of screening for comorbid sleep disorders in patients with ADHD symptoms. Special care must be taken in future studies to ensure that such comorbidities are accounted for when analyzing the sleep of children with ADHD. The analysis of the microarchitecture of sleep with enhanced polysomnographic biomarkers may prove useful in identifying sleep characteristics unique to children with ADHD.

In this large sample of children with ADHD, parents and clinicians may find it reassuring that stimulant prescription was not correlated with any decrease in sleep quality. Sleep disturbances secondary to stimulant prescription in children with ADHD may occur in individuals on a case-to-case basis. Changing the type of medication, its timing, or its dosage may be worth trying prior to stopping stimulants altogether. While stimulant prescription in children with OSA may help with daytime hypersomnolence and attention deficits, it does not alleviate their AHI or sleep fragmentation, and therefore does not protect children from the long-term cardiometabolic consequences of OSA. Starting stimulant treatment in children prior to screening for other sleep disorders may mask comorbid sleep disorder symptoms and delay their diagnosis and treatment.

Unlike with stimulants, prescription of SSRI/SNRIs demonstrated increased sleep fragmentation in children with ADHD without comorbid OSA. SSRI/SNRIs are often used to treat psychiatric illnesses commonly seen in ADHD. More research into SSRI/SNRI use in the ADHD population may prove helpful in determining whether this increased sleep fragmentation is associated with worse daytime ADHD symptoms.

## Data Availability

The raw data supporting the conclusions of this article will be made available by the authors, without undue reservation.
