# Supplementary material for: Polysomnographic insights into the attention-deficit/hyperactivity disorder and obstructive sleep apnea connection in children
Source: Front Sleep. 2024 Oct 7;3:1451869. doi: 10.3389/frsle.2024.1451869 (PMC12713972; doi:10.3389/frsle.2024.1451869)
Supplement: Supplementary file 1 [file Table_1.DOCX]

**SUPPLEMENTAL FIGURES:**

|  | **ADHD+**/**AHI <1** | **ADHD+**/**AHI ≥ 1** | p-value |
| --- | --- | --- | --- |
| N= | 133 | 311 |  |
| Arousal index | 11.2 (5.8) | 16.7 (11.1) | <.0001 |
| Sleep efficiency | 79.4 (13.8) | 80.4 (12.5) | .4268 |
| % REM sleep | 16.2 (9.2) | 19.0 (14.4) | .0403 |
| AHI | 0.4 (0.3) | 7.3 (11.8) | <.0001 |
| Saturation | 97.3 (1.1) | 96.4 (1.4) | <.0001 |
| CO2 | 43.3 (3.8) | 43.1 (3.4) | .6289 |
| PLM index | 1.7 (4.8) | 2.1 (5.8) | .5051 |
| Elevated PLMs | 9 (6.8) | 36 (11.6) | .1241 |

**Supplemental table 1. PSG parameters in children with ADHD and with AHI <1 vs. with ADHD and AHI ≥ 1**

|  | **ADHD+**/OSA- | **ADHD+**/**OSA+** | p-value |
| --- | --- | --- | --- |
| Stimulant usage | 140 (42.0%) | 52 (46.9%) | .3762 |
| Pertinent medications (at the time of sleep study):  Stimulants  SSRI/SNRI  Antihistamines  Iron supplement  Benzo  Melatonin  Antipsychotics  Clonidine  Guanfacine  Anticonvulsants  Other medications which may influence sleep | 140 (42.0%)  94 (28.2%)  96 (28.8%)  37 (11.1%)  8 (2.4%)  76 (22.8%)  37 (11.1%)  51 (15.3%)  47 (14.1%)  62 (18.6%)  41 (12.3%) | 52 (46.9%)  31 (27.9%)  35 (31.5%)  11 (9.9%)  2 (1.8%)  26 (23.4%)  10 (9.0%)  11 (9.9%)  18 (16.2%)  13 (11.7%)  16 (14.4%) | .3762  .9514  .5887  .7241  .7119  .8964  .5330  .1548  .5874  .0926  .5664 |

**Supplemental table 2. Differences in stimulant and psychoactive medication prescription in patients with ADHD, with and without OSA**

|  | **ADHD+**/OSA-  **SSRI/SNRI+** | **ADHD+**/OSA-SSRI/SNRI - | p-values |
| --- | --- | --- | --- |
| N= | 94 | 239 |  |
| Arousal index | 13.6 (6.9) | 11.6 (5.4) | .0061 |
| Sleep efficiency | 79.7 (13.3) | 81.0 (12.6) | .4056 |
| % REM sleep | 15.9 (8.9) | 18.6 (13.1) | .0702 |
| AHI | 1.5 (1.2) | 1.5 (1.1) | .8349 |
| Average saturations | 96.7 (1.3) | 97.0 (1.3) | .0719 |
| Average CO2 | 42.7 (3.7) | 43.5 (3.5) | .1404 |
| PLM index | 2.5 (6.7) | 1.5 (4.7) | .1504 |
| Elevated PLM index | 11 (11.7) | 19 (8.0) | .2817 |

**Supplemental table 3a. PSG sleep parameters in patients with ADHD without OSA, with and without SSRI/SNRI prescription**

|  | **ADHD+**/**OSA+**  **SSRI/SNRI+** | **ADHD+**/**OSA+**  SSRI/SNRI - | p-values |
| --- | --- | --- | --- |
| N= | 31 | 80 |  |
| Arousal index | 25.0 (11.7) | 23.2 (15.2) | .5984 |
| Sleep efficiency | 75.4 (13.8) | 79.9 (12.8) | .0863 |
| % REM sleep | 19.8 (16.9) | 19.2 (15.4) | .8561 |
| AHI | 15.1 (13.4) | 17.0 (17.1) | .5926 |
| Average saturations | 95.7 (1.7) | 96.0 (1.1) | .2560 |
| Average CO2 | 43.0 (2.1) | 43.2 (3.8) | .8360 |
| PLM index | 3.5 (8.0) | 2.0 (5.0) | .2255 |
| Elevated PLM index | 5 (16.1) | 10 (12.5) | .5989 |

**Supplemental table 3b. PSG sleep parameters in patients with ADHD and OSA, with and without SSRI/SNRI prescription**
